# Using clinical cases with diagnostic errors and malpractice claims: impact on anxiety and diagnostic performance in GP clinical reasoning education

**DOI:** 10.1007/s10459-025-10412-z

**Published:** 2025-02-03

**Authors:** Charlotte van Sassen, Silvia Mamede, Jacky Hooftman, Walter van den Broek, Patrick Bindels, Laura Zwaan

**Affiliations:** 1 Department of General Practice, Erasmus MC, University Medical Center Rotterdam, Rotterdam, The Netherlands; 2 Institute of Medical Education Research Rotterdam (iMERR), Erasmus MC, University Medical Center Rotterdam, Rotterdam, The Netherlands; 3 Department of Psychology, Education and Child Studies, Erasmus School of Social and Behavioral Sciences, Rotterdam, the Netherlands; 4 Department of Public and Occupational Health, Amsterdam UMC, Vrije Universiteit, Amsterdam, The Netherlands; 5 Amsterdam Public Health Institute, Quality of Care, Amsterdam, The Netherlands

**Keywords:** Diagnostic errors, Clinical reasoning education, Malpractice claim, Primary care, Emotions, Diagnostic performance

## Abstract

Erroneous and malpractice claim cases reflect knowledge gaps and complex contextual factors. Incorporating such cases into clinical reasoning education (CRE) may enhance learning and diagnostic skills. However, they may also elicit anxiety among learners, potentially impacting learning. As a result, the optimal utilization of such cases in CRE remains uncertain. This study aims to investigate the effect of erroneous and malpractice claim case vignettes on anxiety and future diagnostic performance in CRE and explores possible underlying factors that may influence learning, including self-reported confidence in the final diagnosis, learners’ satisfaction, and retrospective impact of the cases. In this three-phase experiment, GP residents and supervisors were randomly assigned to one of three experimental conditions: neutral (without reference to an error), erroneous (involving a diagnostic error), or malpractice claim (involving a diagnostic error along with a malpractice claim description). During the first session, participants reviewed six cases exclusively in the version of their assigned condition, with anxiety levels measured before and after. In the second session, participants solved six neutral clinical cases featuring the same diagnoses as those in the learning phase but presented in different scenarios, along with four filler cases. Diagnostic performance and self-reported confidence in the diagnosis were assessed. The third session measured learners’ satisfaction and longer-term impact on the participants. Case vignettes featuring diagnostic errors or malpractice claims did not lead to increased anxiety and resulted in similar future diagnostic performance compared to neutral vignettes. Additionally, self-reported confidence, learners’ satisfaction and long-term impact scores did not differ significantly between conditions. This suggests these cases can be integrated into CRE programs, offering a valuable source of diverse, context-rich examples that broaden case libraries without interfering with diagnostic performance or causing anxiety in learners.

**Supplementary Information:**

The online version contains supplementary material available at 10.1007/s10459-025-10412-z.

## Introduction

Diagnostic errors, defined as missed, delayed, or incorrect diagnoses (Graber et al., 2005), account for the majority of malpractice claims in the United States and Europe (Fernholm et al., 2019; Saber Tehrani et al., 2013; Wallace et al., 2013). Many of the diagnosis-related malpractice claims show that errors occur when patients present atypically or have complex contextual factors, resulting in physicians failing to recognize the disease (van Sassen et al., 2022). To improve the ability to recognize atypical presentations, improving clinical reasoning education (CRE) is important (Graber et al., 2018; National Academies of Sciences Engineering & Medicine, 2015). In a previous study, we proposed the use of malpractice cases as clinical vignettes because of their potential to highlight knowledge gaps with implications for patient care (van Sassen et al., 2022). Incorporating malpractice cases into CRE could enhance the development of illness scripts (Charlin et al., 2000, 2007; Lubarsky et al., 2015) for high-risk diseases by highlighting subtle differences between diseases, their presentations, and real-world contextual complexities that influence diagnostic decision making. These complexities include factors such as patient perspectives, the doctor-patient relationship, access to diagnostic tests, support staff, and the healthcare environment. Such scenarios can emphasize the importance of considering contextual complexity in clinical reasoning (Durning et al., 2020; Merkebu et al., 2020). By providing a more authentic learning experience compared to fictional cases, malpractice cases may improve diagnostic reasoning skills and potentially reduce diagnostic errors.

However, the optimal utilization of such vignettes in CRE remains unclear. Many of our residents indicate that reading about a diagnostic error, and more strongly, knowing that the error resulted in a malpractice claim, made them remember the case better. Although the use of malpractice cases in medical education has been suggested by various researchers (Fischer et al., 2006; Meydan, 2014; Tolks et al., 2020), there are no studies that have determined the value of malpractice cases as vignettes for improving clinical reasoning education.

Students and residents report mixed feelings about the use of error and malpractice cases in medical education. On the one hand, they state that they learn well from medical errors and are more likely to remember actual errors than near misses. They are particularly impressed by medical errors that cause serious harm to the patient (Fischer et al., 2006). On the other hand, medical students express lower levels of acceptance and subjective learning outcomes when working with incorrect case examples compared with correct examples (Kopp et al., 2009a, 2009b). Interviews indicate that almost all students and residents view medical errors as worrisome and inevitable (Fischer et al., 2006). Students and residents can experience feelings of guilt, anxiety, anger, and insecurity when exposed to erroneous cases (Fischer et al., 2006; Kiesewetter et al., 2018; Muller & Ornstein, 2007) and seventy percent of residents felt less joy in learning because of concerns about malpractice (Rodriguez et al., 2007). Some even describe committing medical errors as ‘one of the scariest things in becoming a doctor’ (Fischer et al., 2006).

Learning from erroneous examples has been reported to improve clinical skills (Domuracki et al., 2015; Dyre et al., 2017) and diagnostic knowledge (Klein et al., 2019; Kopp et al., 2008, 2009a, 2009b; Stark et al., 2011) in students. However, texts can elicit emotions even when the reader is not directly involved in the scenarios (Pekrun, 2022). The real-world context of the malpractice and erroneous cases is anticipated to trigger emotional responses from participants. These reactions are likely influenced by participants' identification with the clinician’s role in the vignette and their understanding of the potential consequences of diagnostic errors. Therefore, reading about an error and a subsequent malpractice claim may trigger intense negative emotions (Kiesewetter et al., 2018; McConnell & Eva, 2012; Ruedinger et al., 2017) that can profoundly affect learning (Pekrun, 2022). Although both positive (Brandt et al., 2013; Kensinger, 2007; McConnell & Eva, 2012; Nadarevic, 2017) and negative (Fraser et al., 2012; Kremer et al., 2019; Schmidt et al., 2017) effects of emotion on learning and memory have been reported in the literature, our previous study found no difference in the diagnostic performance of GP residents for erroneous case vignettes derived from a claims database ending either with or without a malpractice claim (Van Sassen et al., 2023). To gain further insights into the role of anxiety in learning and diagnostic performance and to corroborate previous findings using a different study design, we investigated whether exposure to one of three versions of case vignettes—namely, neutral cases (N), cases with diagnostic errors (E), and cases with diagnostic errors resulting in malpractice claims (M)—affected actual anxiety levels and future diagnostic performance in GP residents and supervisors in CRE. To explore additional factors that may influence learning from different case scenarios in clinical reasoning education, we compared participants' self-reported confidence in their final diagnosis and the retrospective impact of the cases on their reflection of the condition and clinical experiences post-exposure. These factors can influence how participants process and integrate new knowledge into their clinical practice, ultimately affecting their learning. Additionally, we assessed participants’ satisfaction with the learning experience, as engagement is a relevant factor that can influence motivation and may contribute to successful learning. This methodology further investigates the effect of erroneous and malpractice claim cases as vignettes for clinical reasoning education.

## Methods

All methods followed relevant guidelines and regulations.

### Participants

Participants were final-year (third-year) residents and supervisors in the three-year GP vocational training at Erasmus Medical Center (Erasmus MC) in Rotterdam and Amsterdam University Medical Center (Amsterdam UMC) in Amsterdam, The Netherlands.

### Setting

Clinical reasoning education (CRE) in the three-year GP training program at Erasmus MC Rotterdam and Amsterdam UMC is delivered through both daily clinical practice and weekly in-person group sessions. These “study group sessions”, consisting of 12–14 residents, provide comprehensive education on all aspects of general practice. The weekly educational sessions are held at the university, led by teaching staff and cover eight core clinical reasoning themes over the course of three years. Each theme includes fictitious clinical case vignettes, often featuring common diagnoses and typical disease presentations to help residents hone their diagnostic skills. In daily clinical practice, third-year residents receive one-on-one supervision from senior GPs, who also attend educational sessions at the university on a monthly basis in similar “study groups” of 12–14 supervisors.

This clinical reasoning experiment was conducted in 2021 and 2022 using Qualtrics (Provo, UT), a web-based survey tool. For residents, the experiment was integrated into their regular weekly educational sessions, during which participants received a link to the online survey. For supervisors, only the first session was conducted during their monthly educational meeting, while the remaining sessions were completed independently.

To minimize bias, participants were kept unaware of the study’s purpose and the connection between sessions 1 and 2. This approach prevented them from realizing that session 2 measured their diagnostic performance for diagnoses they had been exposed to in session 1. Randomization into one of the three conditions was done per study group to limit interactions between participants assigned to different conditions across sessions. The study groups were at similar phases of training and composed randomly. At Erasmus MC, participants were randomized into all three conditions. However, due to a limited number of participants, residents from Amsterdam UMC and supervisors from Erasmus MC were randomized into only the neutral and malpractice conditions.

### Study design

**Fig. 1:**
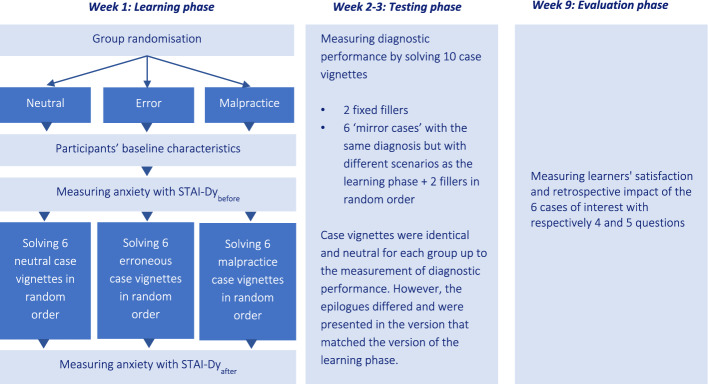
Study design

In this three-phase, between-subjects experiment, participants were assigned to one of three experimental conditions. In the first session (learning phase), participants solved six cases, all presented in either a neutral, erroneous, or malpractice claim version. Anxiety levels were measured before and after reviewing the cases. In the second session (testing phase), participants solved 10 neutral cases: 4 fillers and 6 ‘mirror cases’: cases with identical diagnoses as the first session but different case vignette scenarios. Diagnostic performance and self-reported confidence in the final diagnosis were measured. In the third session (evaluation phase), overall satisfaction with the learning materials and the retrospective impact of the cases on the participants were measured (see Fig. 1).

### Materials

#### Claims database and case development

The largest liability insurer in the Netherlands provided anonymized data on diagnostic errors from 2012 to 2017 for educational and research purposes. Although the data were mined by the insurance company, they had no influence on any aspect of the research.

Twelve different cases covering six diagnoses (tendon rupture, arterial occlusion, retinal detachment, cerebrovascular accident, deep vein thrombosis, and fracture) were selected from the claims database based on the educational priorities of a previous study (van Sassen et al., 2022). For each of the six diagnoses, two cases with the same diagnosis but with different scenarios were selected (‘mirror cases’), one of which was used for the learning phase and one for the testing phase. The mirror cases were intentionally selected to include some overlap in atypical features or diagnostic challenges, ensuring that learning from one case could potentially enhance diagnostic reasoning in the other. The corresponding anonymized files were retrieved from the insurance company and summarized into clinical vignettes. All twelve cases were written in a neutral, erroneous, and malpractice version, all in the same structure and with the same clinical and patient information. In the error version, erroneous decisions were shown with a description of the consequences for the patient. The cause of this error was a cognitive error, such as cognitive bias (e.g.. availability bias, confirmation bias), omissions (e.g., failure to consider certain symptoms), misinterpretation (e.g., misreading clinical signs), or knowledge gaps (e.g., lack of familiarity with certain conditions), as these are more relevant to the CR process than, for example, system-related errors. The malpractice version added that the error resulted in a malpractice claim for which liability was accepted and compensation was paid. To avoid any learning effects related to the length of the case vignette, the additional information in the error and malpractice cases was replaced by general medical theorical information about the disease in the neutral and error cases in order to achieve the same word count (see for examples of clinical case vignettes in various versions Appendix 1). In addition, four fictitious filler clinical case vignettes, also written in three versions, with different diagnoses were developed (only for the second session).

#### State-trait anxiety inventory

Participants’ anxiety levels before and after reading the cases were assessed using the validated Dutch translation of the State-Trait Anxiety Inventory (STAI) (Defares et al., 1980; Spielberger et al., 1983; Van der Ploeg, 1982). Since the focus was on participants’ anxiety during the experiment, only the first part of the STAI-DY, which measures state anxiety, was used.

The STAI is a reliable and valid tool for assessing both state and trait anxiety, with excellent internal consistency among various populations, including working adults and students (Spielberger et al., 1983). It has been widely used in medical education research, particularly in simulation-based and clinical reasoning education, across a range of learners from students and residents to professionals (Dunphy et al., 2010; LeBlanc et al., 2012; Peterlini et al., 2002; P. Pottier et al., 2013; Pottier et al., 2015; Tjønnås et al., 2024).

The state anxiety portion of the STAI-DY comprises 20 items rated on a 4-point Likert scale, ranging from 1 (not at all) to 4 (very much). Final STAI-DY scores range from 20 to 80, with mean scores for Dutch students reported as 34.3 (SD 8.3) for males and 35.2 (SD 8.4) for females (Van der Ploeg, 1982). Anxiety levels are typically classified as “no or low anxiety” (20–37), “moderate anxiety” (38–44), and “high anxiety” (45–80) (Kayikcioglu et al., 2017).

### Procedure

#### First session: learning phase: participant characteristics and anxiety levels

In the first session (week 1), participants’ pre-exposure anxiety levels were measured using STAI-DY after obtaining informed consent and participant characteristics (see Appendix 2). Participants then solved six clinical vignettes in a random order, all in the same experimental condition (neutral, error, malpractice) according to their randomization. The vignettes followed a serial cue method (Schmidt et al., 2015): participants received initial case information and responded to various questions, such as those pertaining to physical examination points of interest and management or diagnostic tests. The various scenarios (neutral, error malpractice) unfolded gradually with the correct diagnosis eventually becoming clear to all participants in every case. Responses to the questions of this task were not analyzed, as the goal of this phase was exposure and learning only. Finally, anxiety levels were measured again with the STAI-DY.

#### Second session: testing phase: diagnostic performance and self-reported confidence

One to two weeks later (weeks 2–3), participants completed six clinical cases featuring the same diagnoses as those in the learning phase but presented in different scenarios (‘mirror cases’). This was done to assess whether their diagnostic performance varied based on prior exposure to the case vignettes under the different experimental conditions (neutral, erroneous, or malpractice). To reduce the likelihood that participants would recognize some of the diagnoses from Session 1, four cases were added as filler cases. The task started with two fixed filler cases to reduce the chance that participants would immediately recognize the same diagnoses from the first session. These were followed by the six ‘mirror’ cases of interest and two remaining filler cases, presented in random order. The first part of the clinical case vignettes was identical for each condition and written in a neutral tone with no reference to errors or malpractice claims. After the first (neutral) part of each case vignette, participants were asked questions measuring diagnostic performance and self-reported confidence: 1. What is your next step?; 2a. What is your most probable diagnosis?; 2b. What is your level of confidence in your diagnosis? (scale 0–100%); 3. What is your differential diagnosis? To take into account the process of arriving at the correct diagnosis, diagnostic performance was measured not only with ‘most probable diagnosis’, but also with the parameters ‘next step’ and ‘differential diagnosis’. Answers could be further specified with free text or multiple choice (see Appendix 3). Only the responses to the six ‘mirror cases’ (cases of interest) were analyzed.

After completing answering all questions measuring diagnostic performance and confidence for all ten cases, participants received epilogues corresponding to their experimental condition in the learning phase. This was done in order to measure retrospective impact in session 3 after repetitive exposure to an experimental condition. The information in the epilogue detailed the GP’s correct (neutral) or incorrect (error and malpractice) decision and its consequences for the patient’s scenario. In the malpractice version, a description of the claim was also included. To balance word count, this additional text of the malpractice version was replaced with medical theoretical information in the neutral and partially erroneous versions in the same manner as in the learning phase.

#### Third session: evaluation phase: learners’ satisfaction and retrospective impact

In the final session, 6 weeks later (week 8–9), participants answered four general questions about their satisfaction with the previous two sessions. They then answered, separately for each of the six cases of interest, more specific questions about the retrospective impact of the cases on their reflection of the condition and their personal clinical experience with that particular disease over the previous six weeks (see Appendix 4).

### Analysis

All calculations were done using SPSS Statistics version 25 for Windows (IBM). Differences were considered significant at *p* < 0.05 level. Analyses were conducted for all participants together, and for supervisors and residents separately.

An a priori power analysis was conducted using G*Power version 3.1.9.6 28 (Faul et al., 2007) to determine the minimum sample size required to test the study hypothesis. Results indicated the required sample size to achieve 80% power for detecting a medium effect, at a significance criterion of α = 0.05, was N = 69 for a repeated measures, between subjects ANOVA (anxiety levels) and N = 90 for a between subjects one-way ANOVA (diagnostic performance/confidence/learners’ satisfaction/ retrospective impact).

#### Participant characteristics and previous working experience

Mean age (SD), gender distribution (%) and previous work experience yes/no (%) were calculated for all participants together and each group separately (residents Erasmus MC, supervisors Erasmus MC, residents Amsterdam UMC). For supervisors, the mean number of years of practice as a general practitioner (range) was also calculated.

#### Anxiety levels (STAI-DY)

After reversing the score for paradoxically formulated items, scores were summed for each participant. Changes in mean anxiety levels before (STAI_before_) and after (STAI_after_) solving the case vignettes were compared between conditions using a between-subjects repeated measures ANOVA. Pairwise comparisons between the conditions were made with Bonferroni adjustment for STAI_before_ and STAI_after_ separately.

#### Diagnostic performance

Two senior staff member GPs, one of whom was involved in this study, developed a detailed assessment rubric for each case and all diagnostic performance questions prior to data collection. This rubric outlined clear criteria for scoring responses on a three-point scale: 1 point for completely correct answers, 0.5 points for partially correct answers or those not specific enough compared to completely correct answers, and 0 points for incorrect or absent answers. Using this rubric, the senior GPs independently assessed the accuracy of participants’ responses to ensure consistent and objective scoring. Interrater reliability was found to be substantial (Landis & Koch, 1977), Kappa = 0.770 (*p* < 0.001), 95% CI (0.723, 0.817) for most probable diagnosis; Kappa = 0.756 (*p* < 0.001), 95% CI (0.697, 0.815) for next step; and Kappa = 0.773 (*p* < 0.001), 95% CI (0.726, 0.820) for differential diagnosis. Disagreements between raters were resolved by discussion. Average scores for the six cases were calculated per participant for all three diagnostic performance parameters. A one-way ANOVA was then performed to compare the mean diagnostic performance scores between the neutral, erroneous, and malpractice groups.

#### Self-reported confidence

After calculating the average scores of the six cases per participant, mean self-reported confidence scores (measured on a scale of 0–100%) for *'What is your most probable diagnosis'* were compared between conditions, using a one-way ANOVA. Pearson's correlation and linear regression were performed between self-reported confidence scores and the score for most probable diagnosis. To assess whether the difference between the Pearson’s correlation coefficients of the conditions was significant, a *z*-value with significance level was calculated using a Fisher *r-to-z* transformation.

#### Learners’ satisfaction and retrospective impact

Mean scores for the four general satisfaction parameters were compared across conditions using a one-way ANOVA. For retrospective impact questions, the average percentage of participants who answered "yes" to the questions over the six cases was calculated, and a one-way ANOVA was performed subsequently to compare the mean percentages across conditions.

## Results

### Participants (first session)

The questionnaires of the three sessions were received by 75 GP residents from Erasmus MC, 28 GP residents from Amsterdam UMC and 81 GP supervisors from Erasmus MC. Respectively 46 (61%), 13 (46%) and 29 (36%) (total n = 88) participants completed both questionnaires of the first and second session and 27 (36%), 0 (0%) and 15 (18.5%) participants completed the questionnaire of the third session, after participating in both previous sessions (total n = 42) (see Appendix 5 for participant inclusion and exclusion). Participant characteristics are presented in Appendix 6. All residents were in their third (final) year of residency, and almost all (96.5%) had previous work experience before starting residency. For supervisors, the mean number of years in general practice was 24.10 years (SD 7.37), with a range of 10–35 years.

### Anxiety levels (STAI-DY) (first session)

**Fig. 2:**
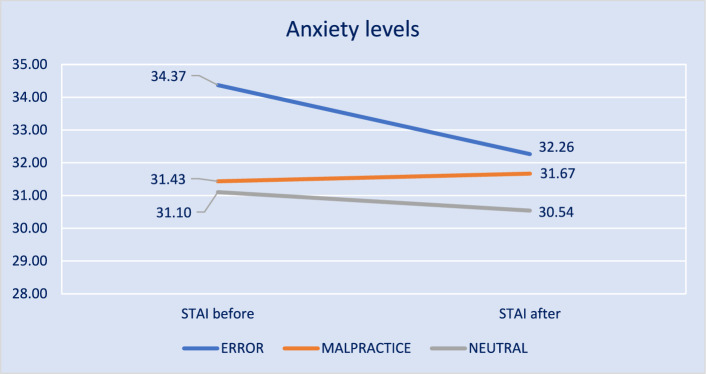
Mean anxiety levels before and after learning task by condition, measured by STAI-DY

The mean STAI-DY scores before and after solving the six vignettes in the first session are shown in Fig. 2. All fall into the “no or low anxiety” category (20–37). The repeated measures between-subjects ANOVA showed no significant differences in change in mean anxiety levels before and after solving the six vignettes across conditions, *F*(2.85) = 0.906 *p* = 0.408. In addition, comparisons of STAI_before_ and STAI_after_ scores between conditions were all non-significant with Bonferroni adjustment (see Appendix 7 for complete table). The results did not change when analyses were performed for the group of supervisors separated from the residents.

### Diagnostic performance (second session)

There were no statistically significant differences in mean diagnostic performance scores between the conditions for any of the diagnostic performance parameters, as determined by one-way ANOVA, either for all participants combined (see Table 1), or for supervisors or residents separately.

**Table 1:** Comparison between conditions of mean scores for parameters of diagnostic performance and self-reported confidence, and Pearson correlation between score for ‘*most probable diagnosis’* and self-reported confidence

Diagnostic Performance Scores (scale 0–1) and Self-Reported Confidence (scale 0–100%)	Neutral n = 39	Error n = 19	Malpractice n = 30	One-way ANOVA *p-value*
	Mean (SD)	Mean (SD)	Mean (SD)	
Next step	0.69 (0.17)	0.74 (0.19)	0.76 (0.14)	0.23^*^
Differential diagnosis	0.62 (0.18)	0.62 (0.11)	0.67 (0.15)	**0.42**^†^
Most probable diagnosis	0.39 (0.15)	0.47 (0.21)	0.41 (0.18)	**0.24**^‡^
Self-reported confidence in most probable diagnosis	55.24 (12.02)	54.59 (9.59)	55.46 (13.10)	**0.97**^§^
Pearson correlation (*r*)/*p*-value between most probable diagnosis and self-reported confidence	0.24 / *p=*0.15	0.42 / *p=*0.075	0.13 / *p=*0.51	

*F(2.85) = 1.506 *p* = 0.23

^†^*F*(2.85) = 0.872 *p* = 0.42

^‡^*F*(2.85) = 1.452 *p* = 0.24

^§^*F*(2.85) = 0.032 *p* = 0.97

### Self-reported confidence (second session)

**Fig. 3:**
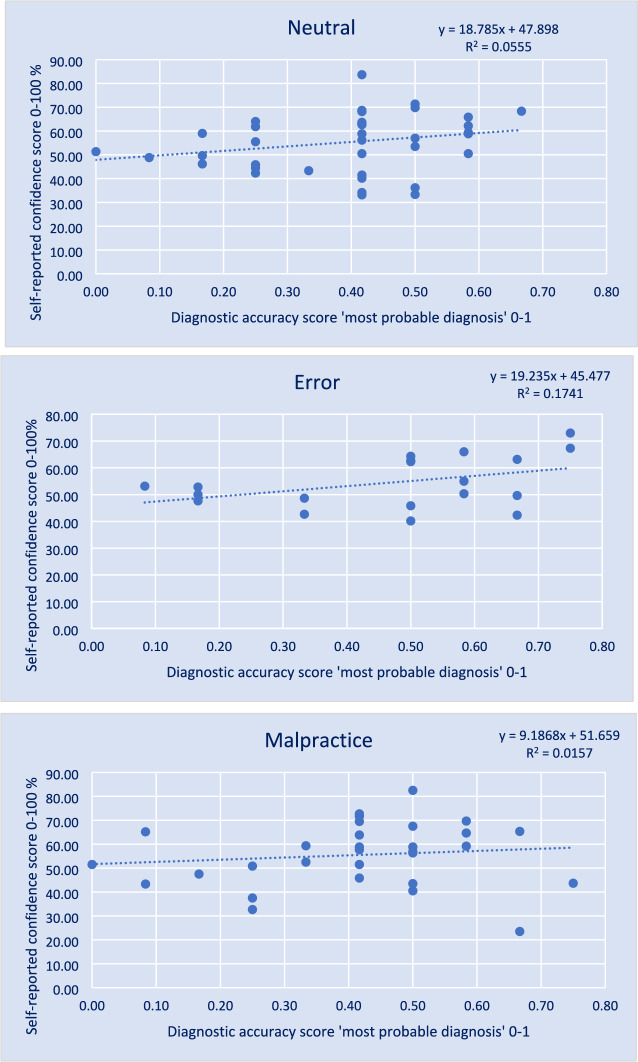
Linear regression of mean diagnostic performance score for ‘*most probable diagnosis’* and self-reported confidence per condition

There was no significant difference in mean self-reported confidence score between conditions. The Pearson correlations between the score for ‘*What is your most likely diagnosis’* and self-reported confidence in that answer were not significant for the conditions (see Table 1 and Fig. 3 for linear regression). There was no significant difference between the correlation coefficients of the three case conditions calculated by Fisher *r*-to-*z* transformation, *r*N-*r*E *z* = -0.68 *p* = 0.4965; *r*N-*r*M *z* = 0.45 *p* = 0.6527; *r*E-*r*M *z* = 1.01 *p* = 0.3125 (two-tailed). The results did not change when supervisors and residents were analyzed separately.

### Learners’ satisfaction and retrospective impact (third session)

Although the mean scores for the four general satisfaction parameters were slightly higher for malpractice and error cases than for neutral cases, the difference between the conditions was not significant (see Table 2). The mean percentage of participants responding ‘yes’ to each parameter of retrospective impact also did not differ significantly between the conditions (see Table 3). There was no change in results when analyses were performed for the group of supervisors separate from residents. We initially planned to perform a qualitative analysis of the free-text responses provided by participants. However, only a small number of participants submitted responses, and the content lacked the depth required for meaningful qualitative analysis. Consequently, we decided not to pursue this analysis further.

**Table 2:** Comparison between conditions of mean scores for general learners’ satisfaction parameters

Learners’ satisfaction (scale 0–100%)^a^	Neutral n = 16	Error n = 14	Malpractice n = 12	One-way ANOVA *p*-value
	Mean (SD)	Mean (SD)	Mean (SD)	
I enjoyed doing this clinical reasoning exercise	72.56 (21.88)	75.64 (8.14)	75.33 (15.37)	0.86^*^
I found it instructive to do this clinical reasoning exercise	64.06 (24.76)	71.93 (11.60)	73.17 (16.56)	**0.38**^†^
I found the cases interesting	71.31 (22.14)	75.79 (9.10)	77.42 (14.20)	**0.59**^‡^
I found it a valuable addition to the educational program	67.75 (25.02)	73.07 (12.44)	70.75 (18.36)	**0.76**^§^

*F(2.39) = 0.158 *p* = 0.86

^†^*F*(2.39) = 0.999 *p* = 0.38

^‡^*F*(2.39) = 0.527 *p* = 0.59

^§^*F*(2.39) = 0.276 *p* = 0.76

^a^Answers on a scale of 0 to 100%, where 0% is ‘I do not agree at all’ and 100% is ‘I agree very much’

**Table 3:** Comparison between conditions of mean percentages of participants responding “Yes” to retrospective impact parameters

Retrospective Impact (Number of participants that answered yes)	Neutral n = 16	Error n = 14	Malpractice n = 12	One-way ANOVA *p* value
	N (%[SD])	N (%[SD])	N (%[SD])	
Have you ever thought about this condition in the past few weeks?	4.17 (0.26 [0.23])	5.17 (0.37 [0.29])	4.00 (0.33 [0.32])	0.56^*^
Have you seen patients in whom you included this condition in the differential diagnosis?	7.17 (0.45 [0.29])	6.33 (0.45 [0.28])	6.00 (0.50 [0.21])	**0.86**^**†**^
Did you discuss this case vignette or this particular condition with your fellow residents or supervisor/ colleague as a result of your participation in the study?	1.50 (0.09 [0.16])	0.33 (0.02 [0.06])	2.33 (0.19 [0.27])	**0.065**^**‡**^
Did your participation in the clinical reasoning training study influence your policy or did you act differently when caring for patients?	1.50 (0.09 [0.15])	1.00 (0.07 [0.09])	2.00 (0.17 [0.26])	**0.35**^**§**^

*F(2.39) = 0.599 *p* = 0.56

^†^*F*(2.39) = 0.151 *p* = 0.86

^‡^*F*(2.39) = 2.926 *p* = 0.065

^§^*F*(2.39) = 1.073 *p* = 0.35

## Discussion

To gain more insight into the effect of erroneous and malpractice claims cases as vignettes for clinical reasoning education, we examined whether exposure to three different types of clinical reasoning cases (neutral, diagnostic error, malpractice claim) influenced anxiety levels and was associated with changes in future diagnostic performance in GP residents and supervisors. Although previous studies have reported that students and residents are affected by diagnostic errors and that malpractice claims in particular evoke emotions such as guilt, fear, anger, and uncertainty (Fischer et al., 2006; Kiesewetter et al., 2018; Muller & Ornstein, 2007), which can influence learning (Pekrun, 2022), we found no evidence of these effects in this study. Erroneous and malpractice claims case vignettes did not seem to affect anxiety levels or diagnostic performance in either GP residents or supervisors compared to neutral case vignettes.

To explore additional underlying factors that may influence the learning process from different case scenarios in clinical reasoning education, we compared self-reported confidence in the final diagnosis and retrospective impact scores. These parameters were examined to determine whether exposure to the various conditions affected participants' confidence in their diagnostic reasoning, clinical discussions, and actions in the weeks following exposure to the cases. We also assessed learners’ satisfaction to evaluate participants’ engagement with the training, as engagement is a relevant factor that can influence motivation and may contribute to successful learning. The low self-reported confidence and poor calibration between self-reported confidence and diagnostic performance found in this study were independent of case condition, but consistent with the literature on confidence and calibration (Berner & Graber, 2008; Costa Filho et al., 2019; Friedman et al., 2005; A. N. Meyer et al., 2013; A. N. D. Meyer & Singh, 2017; Omron et al., 2018). Overall learners’ satisfaction with the sessions did not differ significantly between conditions and did not show lower acceptability for cases involving a diagnostic error or malpractice claim in our study. This contrasts with studies that found lower acceptability for error cases in medical students (Kopp et al., 2009a, 2009b). Although Kopp et al.'s study focused on a different learner population (students rather than residents), the comparison is relevant for understanding how different types of cases are perceived in educational contexts. Finally, retrospective impact parameters also showed similar scores for all case conditions, in contrast to the finding that students and residents report being more impressed by medical errors that cause serious harm and are more likely to remember the actual error than near misses (Fischer et al., 2006).

One possible explanation for the lack of differences across parameters is that, although the literature indicates that texts can evoke emotions in readers (Pekrun, 2022), participants may not have found the paper-based case vignettes as engaging or impactful as real-life cases, which could result in a lower emotional response. Paper-based cases might lack the vividness and depth of a personal narrative about a malpractice incident, which could evoke stronger emotional reactions. Additionally, the task in this study focused solely on diagnostic questions—such as identifying the next diagnostic step, listing a differential diagnosis, and selecting the most probable diagnosis—without asking questions about the management of the error itself or the consequences for the patient, such as handling the patient’s emotions, disclosure, or communicating with family. These aspects are known to evoke more intense emotional responses in physicians. However, research by Peabody has shown that paper cases are a valid method for assessing physicians' competence, suggesting they remain a reliable tool for evaluating diagnostic reasoning (Peabody et al., 2000, 2004).

Another explanation for the lack of differences may be that that experienced students, such as our third-year GP trainees and supervisors, who already have extensive experience with emotionally arousing situations, have been taught to manage their emotions during their medical training (LeBlanc et al., 2015). Because of this, and the fact that advanced learners have already mastered the basics, they may be able to handle more cognitive load, which is reflected in equivalent diagnostic performance scores.

Given the lack of differences in any of the endpoint parameters between the different conditions, it appears that advanced learners may not perceive malpractice claim cases differently from erroneous or neutral cases in their learning process. While our findings suggest that emotions may not play a significant role in CRE with malpractice cases in advanced learners, the learning process and the type of information remembered may still differ between case conditions, warranting further investigation. Previous studies have found that the processing of emotional information comes (partially) at the expense of clinical information (Mamede et al., 2017; Schmeichel, 2007; Schmeichel et al., 2003), contributing to increased extrinsic cognitive load. However, this may not have affected the diagnostic performance of our advanced participants, as their expertise might have enabled them to handle the additional cognitive demands more effectively. Studies showing that learners with solid knowledge benefit from incorrect examples, support this theory (Große & Renkl, 2004, 2007). Further research may reveal how different types of information (e.g., claim-specific vs. case-specific) are cognitively processed when malpractice claims are used for educational purposes, particularly in terms of how claim-specific details are incorporated into diagnostic frameworks. Understanding these processes could help educators design learning experiences that enhance illness script development, diagnostic vigilance, and adaptive expertise, ultimately supporting trainees in navigating complex clinical scenarios.

Despite the need for further exploration, the use of malpractice claims in CRE remains valuable, as these cases often highlight knowledge gaps and provide diverse, context-rich examples (van Sassen et al., 2022), with our study suggesting that diagnostic performance is not negatively affected. However, it is essential to ensure that incorporating malpractice claims in education does not encourage defensive medicine and subsequent overdiagnosis and overtreatment, and therefore we recommend using them in combination with neutral case vignettes.

### Limitations

This study was conducted in the general practice departments of two academic universities in the Netherlands, where residents are relatively well trained in clinical reasoning. This may limit generalizability to sites in other parts of the world with less time devoted to clinical reasoning skills. In addition, this study did not take into account cultural differences in dealing with diagnostic errors and claims and the emotions involved.

Another limitation is the challenge of measuring the impact of reading about errors and claims on clinical practice. Although we attempted to assess this during the evaluation phase by inquiring about the retrospective impact of the cases on participants' thoughts about the disease and their personal experiences with it, this subjective assessment may not fully capture the actual impact on practice. Additionally, the timeframe of 9 weeks after exposure to diagnostic errors and malpractice claims may be too short if GPs did not encounter a similar diagnosis during that period. While a qualitative approach to the free-text responses added to the answers of the questions could provide deeper insights into the retrospective impact, few participants opted to provide free-text responses, and the text provided was not rich enough to allow for a meaningful qualitative analysis. However, this could be an interesting focus for future research.

In our study design, we replaced additional information related to the error and malpractice claim with theoretical information in the neutral cases to maintain a consistent word count across vignettes, thereby minimizing any unintended learning effects from variations in vignette length. Although this theoretical information is neutral and lacks emotional content, which minimizes its potential as a confounding factor for anxiety levels, its informative nature could still introduce a learning effect that might influence diagnostic performance.

Randomization was done per study group in order to limit interactions between participants of different conditions between the sessions. However, controlling interactions between sessions was not feasible. Additionally, there could be chance variations in group levels between groups, which could not be corrected for.

The study had a low response rate, with only 88 out of 184 participants completing the first two sessions due to absences from the weekly educational sessions. We lack details regarding the causes of absences due to privacy regulations, but they were not different from regular absence rates. Considering that the study was integrated into a standard educational session, it's probable that absences were a result of reasons such as vacation or illness rather than being related to the study itself, as participants were not informed about it beforehand. Additionally, participants were required to attend both session 1 and 2 to be eligible for inclusion; thus, missing even one session of the first two resulted in exclusion. Furthermore, due to an insufficient number of participants available to form an error group among the residents of Amsterdam UMC and the supervisors of Erasmus MC, the group sizes and compositions across the different conditions became unequal. Finally, power calculations indicated that a sample size of 90 was needed for adequate power to assess diagnostic performance, confidence, learners’ satisfaction and retrospective impact. However, the low response rate, particularly for learners’ satisfaction and retrospective impact (42 participants), limited statistical power for these outcomes. For diagnostic performance and confidence, the sample size (88 participants) nearly met the threshold for statistical power. The small and inconsistent differences observed across conditions, combined with prior research showing no effect on diagnostic accuracy, suggest that small deviations are unlikely to significantly alter the robustness of our conclusions for diagnostic performance and confidence.

## Conclusion and recommendations

Case vignettes featuring diagnostic errors or malpractice claims did not lead to increased anxiety and resulted in similar future diagnostic performance compared to neutral vignettes in clinical reasoning education (CRE). Additionally, no significant differences were found in self-reported confidence, learners’ satisfaction, or the retrospective perceived impact of the cases among third-year GP residents and supervisors. Given these findings, incorporating information about malpractice claims into case vignettes may enrich the clinical reasoning curriculum for advanced learners without negatively impacting the studied parameters. Prior research has demonstrated that such cases offer valuable opportunities for learners to recognize atypical disease presentations and understand the role of context in diagnostic decisions (van Sassen et al., 2022). To minimize potential defensive diagnostic behaviors that errors or malpractice claims might trigger, a balanced inclusion of error-based and neutral vignettes is recommended. Further research should investigate whether cognitive processing varies depending on the type of information presented in different case versions.

## Supplementary Information

Below is the link to the electronic supplementary material.

Supplementary file1 (DOCX 62 kb)

## Data Availability

No datasets were generated or analysed during the current study.

## References

[CR1] Berner, E. S., & Graber, M. L. (2008). Overconfidence as a cause of diagnostic error in medicine. *The American Journal of Medicine,**121*(5), S2–S23. 10.1016/j.amjmed.2008.01.001 10.1016/j.amjmed.2008.01.001 18440350

[CR2] Brandt, K. R., Nielsen, M. K., & Holmes, A. (2013). Forgetting emotional and neutral words: An ERP study. *Brain Research,**1501*, 21–31. 10.1016/j.brainres.2013.01.019 23337616 10.1016/j.brainres.2013.01.019

[CR3] Charlin, B., Tardif, J., & Boshuizen, H. P. A. (2000). Scripts and medical diagnostic knowledge: Theory and applications for clinical reasoning instruction and research. *Academic Medicine*, *75*(2), 182–190 10693854 10.1097/00001888-200002000-00020

[CR4] Charlin, B., Boshuizen, H. P. A., Custers, E. J., & Feltovich, P. J. (2007). Scripts and clinical reasoning: Clinical expertise. *Medical Education,**41*(12), 1178–1184. 10.1111/j.1365-2923.2007.02924.x 18045370 10.1111/j.1365-2923.2007.02924.x

[CR5] Costa Filho, G. B., Moura, A. S., Brandão, P. R., Schmidt, H. G., & Mamede, S. (2019). Effects of deliberate reflection on diagnostic accuracy, confidence and diagnostic calibration in dermatology. *Perspectives on Medical Education,**8*(4), 230–236. 10.1007/S40037-019-0522-5 31290117 10.1007/s40037-019-0522-5 PMC6684533

[CR6] Defares, P. B., van der Ploeg, H. M., & Spielberger, C. D. (1980). *Handleiding bij de Zelf-beoordelings Vragenlijst ZBV. Een nederlandstalige bewerking van de Spielberger State-Trait Anxiety Inventory*. Swets & Zeitlinger.

[CR7] Domuracki, K., Wong, A., Olivieri, L., & Grierson, L. E. M. (2015). The impacts of observing flawed and flawless demonstrations on clinical skill learning. *Medical Education,**49*(2), 186–192. 10.1111/medu.12631 25626749 10.1111/medu.12631

[CR8] Dunphy, B. C., Cantwell, R., Bourke, S., Fleming, M., Smith, B., Joseph, K. S., & Dunphy, S. L. (2010). Cognitive elements in clinical decision-making: Toward a cognitive model for medical education and understanding clinical reasoning. *Advances in Health Sciences Education,**15*(2), 229–250. 10.1007/s10459-009-9194-y 19763856 10.1007/s10459-009-9194-y

[CR9] Durning, S., Holmboe, E., & Graber, M. L. (2020). Special issue: Situativity: A family of social cognitive theories for clinical reasoning and error. *Diagnosis,**7*(3), 1–4. 10.1515/dx-2020-frontmatter3 32924378 10.1515/dx-2019-0100

[CR10] Dyre, L., Tabor, A., Ringsted, C., & Tolsgaard, M. G. (2017). Imperfect practice makes perfect: Error management training improves transfer of learning. *Medical Education,**51*(2), 196–206. 10.1111/medu.13208 27943372 10.1111/medu.13208

[CR11] Faul, F., Erdfelder, E., Lang, A.-G., & Buchner, A. (2007). G*Power 3: A flexible statistical power analysis program for the social, behavioral, and biomedical sciences. *Behavior Research Methods,**39*(2), 175–191. 10.3758/BF03193146 17695343 10.3758/bf03193146

[CR12] Fernholm, R., Pukk Härenstam, K., Wachtler, C., Nilsson, G. H., Holzmann, M. J., & Carlsson, A. C. (2019). Diagnostic errors reported in primary healthcare and emergency departments: A retrospective and descriptive cohort study of 4830 reported cases of preventable harm in Sweden. *European Journal of General Practice,**25*(3), 128–135. 10.1080/13814788.2019.1625886 31257959 10.1080/13814788.2019.1625886 PMC6713141

[CR13] Fischer, M. A., Mazor, K. M., Baril, J., Alper, E., DeMarco, D., & Pugnaire, M. (2006). Learning from mistakes: Factors that influence how students and residents learn from medical errors. *Journal of General Internal Medicine,**21*(5), 419–423. 10.1111/j.1525-1497.2006.00420.x 16704381 10.1111/j.1525-1497.2006.00420.x PMC1484785

[CR14] Fraser, K., Ma, I., Teteris, E., Baxter, H., Wright, B., & McLaughlin, K. (2012). Emotion, cognitive load and learning outcomes during simulation training: Emotion and cognitive load during simulation. *Medical Education,**46*(11), 1055–1062. 10.1111/j.1365-2923.2012.04355.x 23078682 10.1111/j.1365-2923.2012.04355.x

[CR15] Friedman, C. P., Gatti, G. G., Franz, T. M., Murphy, G. C., Wolf, F. M., Heckerling, P. S., et al. (2005). Do physicians know when their diagnoses are correct? Implications for decision support and error reduction. *Journal of General Internal Medicine,**20*(4), 334–339. 10.1111/j.1525-1497.2005.30145.x 15857490 10.1111/j.1525-1497.2005.30145.x PMC1490097

[CR16] Graber, M. L., Franklin, N., & Gordon, R. (2005). Diagnostic Error in Internal Medicine. *Archives of Internal Medicine,**165*(13), 1493. 10.1001/archinte.165.13.1493 16009864 10.1001/archinte.165.13.1493

[CR17] Graber, M. L., Rencic, J., Rusz, D., Papa, F., Croskerry, P., Zierler, B., et al. (2018). Improving diagnosis by improving education: A policy brief on education in healthcare professions. *Diagnosis,**5*(3), 107–118. 10.1515/dx-2018-0033 30145580 10.1515/dx-2018-0033

[CR18] Große, C. S., & Renkl, A. (2004). Learning from worked examples: What happens if errors are included, pp. 356–364.

[CR19] Große, C. S., & Renkl, A. (2007). Finding and fixing errors in worked examples: Can this foster learning outcomes? *Learning and Instruction,**17*(6), 612–634. 10.1016/j.learninstruc.2007.09.008

[CR20] Kayikcioglu, O., Bilgin, S., Seymenoglu, G., & Deveci, A. (2017). State and trait anxiety scores of patients receiving intravitreal injections. *Biomedicine Hub,**2*(2), 1–5. 10.1159/000478993 10.1159/000478993 PMC6945947 31988910

[CR21] Kensinger, E. A. (2007). Negative emotion enhances memory accuracy: behavioral and neuroimaging evidence. *Current Directions in Psychological Science,**16*(4), 213–218. 10.1111/j.1467-8721.2007.00506.x

[CR22] Kiesewetter, I., Könings, K. D., Kager, M., & Kiesewetter, J. (2018). Undergraduate medical students’ behavioural intentions towards medical errors and how to handle them: A qualitative vignette study. *British Medical Journal Open,**8*(3), e019500. 10.1136/bmjopen-2017-019500 10.1136/bmjopen-2017-019500 PMC5857650 29540413

[CR23] Klein, M., Otto, B., Fischer, M. R., & Stark, R. (2019). Fostering medical students’ clinical reasoning by learning from errors in clinical case vignettes: Effects and conditions of additional prompting procedures to foster self-explanations. *Advances in Health Sciences Education,**24*(2), 331–351. 10.1007/s10459-018-09870-5 30627833 10.1007/s10459-018-09870-5

[CR24] Kopp, V., Stark, R., & Fischer, M. R. (2008). Fostering diagnostic knowledge through computer-supported, case-based worked examples: Effects of erroneous examples and feedback. *Medical Education,**42*(8), 823–829. 10.1111/j.1365-2923.2008.03122.x 18564096 10.1111/j.1365-2923.2008.03122.x

[CR25] Kopp, V., Stark, R., Heitzmann, N., & Fischer, M. R. (2009a). Self-regulated learning with case-based worked examples: Effects of errors. *Evaluation & Research in Education,**22*(2–4), 107–119. 10.1080/09500790903494518

[CR26] Kopp, V., Stark, R., Kühne-Eversmann, L., & Fischer, M. R. (2009b). Do worked examples foster medical students’ diagnostic knowledge of hyperthyroidism?: Worked examples improve diagnostic knowledge. *Medical Education,**43*(12), 1210–1217. 10.1111/j.1365-2923.2009.03531.x 19930513 10.1111/j.1365-2923.2009.03531.x

[CR27] Kremer, T., Mamede, S., van den Broek, W. W., Schmidt, H. G., Nunes, M. D. P. T., & Martins, M. A. (2019). Influence of negative emotions on residents’ learning of scientific information: an experimental study. *Perspectives on Medical Education,**8*(4), 209–215. 10.1007/s40037-019-00525-8 31338789 10.1007/s40037-019-00525-8 PMC6684560

[CR28] Landis, J. R., & Koch, G. G. (1977). The measurement of observer agreement for categorical data. *Biometrics,**33*(1), 159–174. 843571

[CR29] LeBlanc, V. R., McConnell, M. M., & Monteiro, S. D. (2015). Predictable chaos: A review of the effects of emotions on attention, memory and decision making. *Advances in Health Sciences Education,**20*(1), 265–282. 10.1007/s10459-014-9516-6 24903583 10.1007/s10459-014-9516-6

[CR30] LeBlanc, V. R., Regehr, C., Tavares, W., Scott, A. K., MacDonald, R., & King, K. (2012). The impact of stress on paramedic performance during simulated critical events. *Prehospital and Disaster Medicine,**27*(4), 369–374. 10.1017/S1049023X12001021 22831965 10.1017/S1049023X12001021

[CR31] Lubarsky, S., Dory, V., Audétat, M.-C., Custers, E., & Charlin, B. (2015). Using script theory to cultivate illness script formation and clinical reasoning in health professions education. *Canadian Medical Education Journal,**6*(2), e61-70. PMC4795084 27004079

[CR32] Mamede, S., Van Gog, T., Schuit, S. C. E., Van den Berge, K., Van Daele, P. L. A., Bueving, H., et al. (2017). Why patients’ disruptive behaviours impair diagnostic reasoning: A randomised experiment. *BMJ Quality & Safety,**26*(1), 13–18. 10.1136/bmjqs-2015-005065 10.1136/bmjqs-2015-005065 26951796

[CR33] McConnell, M. M., & Eva, K. W. (2012). The role of emotion in the learning and transfer of clinical skills and knowledge. *Academic Medicine,**87*(10), 1316–1322. 10.1097/ACM.0b013e3182675af2 22914515 10.1097/ACM.0b013e3182675af2

[CR34] Merkebu, J., Battistone, M., McMains, K., McOwen, K., Witkop, C., Konopasky, A., et al. (2020). Situativity: A family of social cognitive theories for understanding clinical reasoning and diagnostic error. *Diagnosis,**7*(3), 169–176. 10.1515/dx-2019-0100 32924378 10.1515/dx-2019-0100

[CR35] Meydan, C. (2014). Risk management—Learning from the mistakes of others: Risk management for medical students. *Journal of Evaluation in Clinical Practice,**20*(4), 505–507. 10.1111/jep.12165 24815360 10.1111/jep.12165

[CR36] Meyer, A. N., Payne, V. L., Meeks, D. W., Rao, R., & Singh, H. (2013). Physicians’ diagnostic accuracy, confidence, and resource requests: A vignette study. *JAMA Internal Medicine,**173*(21), 1952–1958. 23979070 10.1001/jamainternmed.2013.10081

[CR37] Meyer, A. N. D., & Singh, H. (2017). Calibrating how doctors think and seek information to minimise errors in diagnosis. *BMJ Quality & Safety,**26*(6), 436–438. 10.1136/bmjqs-2016-006071 10.1136/bmjqs-2016-006071 27672123

[CR38] Muller, D., & Ornstein, K. (2007). Perceptions of and attitudes towards medical errors among medical trainees. *Medical Education,**41*(7), 645–652. 10.1111/j.1365-2923.2007.02784.x 17614884 10.1111/j.1365-2923.2007.02784.x

[CR39] Nadarevic, L. (2017). Emotionally enhanced memory for negatively arousing words: Storage or retrieval advantage? *Cognition and Emotion,**31*(8), 1557–1570. 10.1080/02699931.2016.1242477 27741932 10.1080/02699931.2016.1242477

[CR40] National Academies of Sciences Engineering and Medicine. (2015). *Improving diagnosis in health care*. National Academies Press. https://books.google.nl/books?hl=en&lr=&id=-fphCwAAQBAJ&oi=fnd&pg=PR1&dq=improving+diagnosis+in+health+care&ots=CsPzsFpJeT&sig=H0InJdR0eS6pmbgRZK9DBD1kkKE. Accessed 8 May 2017.

[CR41] Omron, R., Kotwal, S., Garibaldi, B. T., & Newman-Toker, D. E. (2018). The diagnostic performance feedback “calibration gap”: Why clinical experience alone is not enough to prevent serious diagnostic errors. *AEM Education and Training,**2*(4), 339–342. 10.1002/aet2.10119 30386846 10.1002/aet2.10119 PMC6194049

[CR42] Peabody, J. W., Luck, J., Glassman, P., Dresselhaus, T. R., & Lee, M. (2000). Comparison of vignettes, standardized patients, and chart abstraction: A prospective validation study of 3 methods for measuring quality. *JAMA,**283*(13), 1715. 10.1001/jama.283.13.1715 10755498 10.1001/jama.283.13.1715

[CR43] Peabody, J. W., Luck, J., Glassman, P., Jain, S., Hansen, J., Spell, M., & Lee, M. (2004). Measuring the quality of physician practice by using clinical vignettes: A prospective validation study. *Annals of Internal Medicine,**141*(10), 771. 10.7326/0003-4819-141-10-200411160-00008 15545677 10.7326/0003-4819-141-10-200411160-00008

[CR44] Pekrun, R. (2022). Emotions in reading and learning from texts: Progress and open problems. *Discourse Processes,**59*(1–2), 116–125. 10.1080/0163853X.2021.1938878

[CR45] Peterlini, M., Tibério, I. F. L. C., Saadeh, A., Pereira, J. C. R., & Martins, M. A. (2002). Anxiety and depression in the first year of medical residency training: Anxiety and depression in the first year of medical residency training. *Medical Education,**36*(1), 66–72. 10.1046/j.1365-2923.2002.01104.x 11849526 10.1046/j.1365-2923.2002.01104.x

[CR46] Pottier, P., Dejoie, T., Hardouin, J. B., Le Loupp, A. G., Planchon, B., Bonnaud, A., & Leblanc, V. R. (2013). Effect of stress on clinical reasoning during simulated ambulatory consultations. *Medical Teacher,**35*(6), 472–480. 10.3109/0142159X.2013.774336 23464842 10.3109/0142159X.2013.774336

[CR47] Pottier, P., Hardouin, J.-B., Dejoie, T., Castillo, J.-M., Le Loupp, A.-G., Planchon, B., et al. (2015). Effect of extrinsic and intrinsic stressors on clinical skills performance in third-year medical students. *Journal of General Internal Medicine,**30*(9), 1259–1269. 10.1007/s11606-015-3314-6 26173521 10.1007/s11606-015-3314-6 PMC4539332

[CR48] Rodriguez, R. M., Anglin, D., Hankin, A., Hayden, S. R., Phelps, M., McCollough, L., & Hendey, G. W. (2007). A longitudinal study of emergency medicine residents’ malpractice fear and defensive medicine. *Academic Emergency Medicine,**14*(6), 569–573. 10.1197/j.aem.2007.01.020 17446194 10.1197/j.aem.2007.01.020

[CR49] Ruedinger, E., Olson, M., Yee, J., Borman-Shoap, E., & Olson, A. P. J. (2017). Education for the next frontier in patient safety: A longitudinal resident curriculum on diagnostic error. *American Journal of Medical Quality,**32*(6), 625–631. 10.1177/1062860616681626 27903769 10.1177/1062860616681626

[CR50] Saber Tehrani, A. S., Lee, H., Mathews, S. C., Shore, A., Makary, M. A., Pronovost, P. J., & Newman-Toker, D. E. (2013). 25-Year summary of US malpractice claims for diagnostic errors 1986–2010: An analysis from the National Practitioner Data Bank. *BMJ Quality & Safety,**22*(8), 672–680. 10.1136/bmjqs-2012-001550 10.1136/bmjqs-2012-001550 23610443

[CR51] Schmeichel, B. J. (2007). Attention control, memory updating, and emotion regulation temporarily reduce the capacity for executive control. *Journal of Experimental Psychology: General,**136*(2), 241–255. 10.1037/0096-3445.136.2.241 17500649 10.1037/0096-3445.136.2.241

[CR52] Schmeichel, B. J., Vohs, K. D., & Baumeister, R. F. (2003). Intellectual performance and ego depletion: Role of the self in logical reasoning and other information processing. *Journal of Personality and Social Psychology,**85*(1), 33–46. 10.1037/0022-3514.85.1.33 12872883 10.1037/0022-3514.85.1.33

[CR53] Schmidt, H. G., van Gog, T., Schuit, S. C. E., van den Berge, K., Van Daele, P. L. A., Bueving, H., et al. (2017). Do patients’ disruptive behaviours influence the accuracy of a doctor’s diagnosis? A randomised experiment: Table 1. *BMJ Quality & Safety,**26*(1), 19–23. 10.1136/bmjqs-2015-004109 10.1136/bmjqs-2015-004109 26951795

[CR54] Schmidt, & Mamede, S. (2015). How to improve the teaching of clinical reasoning: a narrative review and a proposal. *Medical Education,**49*(10), 961–973. 10.1111/medu.12775 26383068 10.1111/medu.12775

[CR55] Spielberger, C. D., Gorsuch, R. L., Lushene, R. E., Vagg, P. R., & Jacobs, G. A. (1983). *Manual for the state-trait anxiety inventory (form Y1–Y2)*. Palo Alto, CA: Consulting Psychologists Press.

[CR56] Stark, R., Kopp, V., & Fischer, M. R. (2011). Case-based learning with worked examples in complex domains: Two experimental studies in undergraduate medical education. *Learning and Instruction,**21*(1), 22–33. 10.1016/j.learninstruc.2009.10.001

[CR57] Tjønnås, M. S., Muller, S., Våpenstad, C., Tjønnås, J., Ose, S. O., Das, A., & Sandsund, M. (2024). Stress responses in surgical trainees during simulation-based training courses in laparoscopy. *BMC Medical Education,**24*(1), 407. 10.1186/s12909-024-05393-3 38610013 10.1186/s12909-024-05393-3 PMC11010405

[CR58] Tolks, D., Kiessling, C., Wershofen, B., Pudritz, Y., Schunk, M., Härtl, A., et al. (2020). Lernen aus Fehlern anhand eines fallbasierten Curriculums im medizinischen Querschnittsbereich Gesundheitssysteme/Gesundheitsökonomie und öffentliche Gesundheitspflege. *Das Gesundheitswesen,**82*(11), 909–914. 10.1055/a-0894-4583 31185501 10.1055/a-0894-4583

[CR59] Van der Ploeg, H. M. (1982). De zelf-beoordelings vragenlijst (STAI-DY). *Tijdschrift Voor Psychiatrie,**24*, 576–588.

[CR60] Van Sassen, C., Mamede, S., Bos, M., Van Den Broek, W., Bindels, P., & Zwaan, L. (2023). Do malpractice claim clinical case vignettes enhance diagnostic accuracy and acceptance in clinical reasoning education during GP training? *BMC Medical Education,**23*(1), 474. 10.1186/s12909-023-04448-1 37365590 10.1186/s12909-023-04448-1 PMC10294315

[CR61] van Sassen, C. G. M., van den Berg, P. J., Mamede, S., Knol, L., Eikens-Jansen, M. P., van den Broek, W. W., et al. (2022). Identifying and prioritizing educational content from a malpractice claims database for clinical reasoning education in the vocational training of general practitioners. *Advances in Health Sciences Education*. 10.1007/s10459-022-10194-8 10.1007/s10459-022-10194-8 PMC10356624 36529764

[CR62] Wallace, E., Lowry, J., Smith, S. M., & Fahey, T. (2013). The epidemiology of malpractice claims in primary care: A systematic review. *British Medical Journal Open,**3*(7), e002929. 10.1136/bmjopen-2013-002929 10.1136/bmjopen-2013-002929 PMC3693415 23869100

