# Supplementary material for: Using clinical cases with diagnostic errors and malpractice claims: impact on anxiety and diagnostic performance in GP clinical reasoning education
Source: Adv Health Sci Educ Theory Pract. 2025 Feb 3;30(5):1403–23. doi: 10.1007/s10459-025-10412-z (PMC12572042; doi:10.1007/s10459-025-10412-z)
Supplement: Supplementary file 1 — Supplementary file1 (DOCX 62 kb) [file 10459_2025_10412_MOESM1_ESM.docx]

**Supplemental Material**

**Appendix 1**

**Example of a clinical case vignette from session 1 on cerebrovascular accident (CVA), presented in three different versions. The questions are designed to stimulate information processing; however, responses are not analyzed in this study. The cases are presented using a serial cue format. Note: the word count was calculated for Dutch texts and may differ in English.**

**CVA, NEUTRAL, 674**

You are on duty at the general practitioner’s out-of-hours service. At 12:45 a.m., the assistant asks you to urgently attend to 31-year-old Mr. Boogaard. Mr. Boogaard’s parents have called the out-of-hours service because he has had a headache since this afternoon, and for the past few hours, he has been unable to move his right arm and leg properly, has difficulty speaking, feels dizzy, and has impaired vision.

Upon arriving at the home of Mr. Boogaard, who lives alone, you meet him and his parents, whom he had alerted. Mr. Boogaard mentions that he is worried he may have had a stroke. He has had a headache all day, feels nauseous, and sometimes vomits a little. Over the course of the afternoon and evening, his symptoms have worsened, and he has noticed a loss of sensation and strength in his right side. He works as an IT specialist and is experiencing a lot of stress at work due to a poor relationship with his supervisor. Mr. Boogaard indicates that he has always been "clumsy" in social interactions.

1. What general questions would you add to the medical history?

- Risk factors: Mr. Boogaard does not smoke, drinks 4 units of alcohol per week, and does not use drugs.
- Medical history: The medical history mentions right-sided tinnitus. Otherwise, Mr. Boogaard has always been healthy.
- Family history: There is no history of cardiovascular diseases or malignancies in the family.

You decide to proceed with a physical examination.

1. What physical examination would you conduct on Mr. Boogaard?

You decide to focus on a general neurological examination.

You find that your interaction with Mr. Boogaard is somewhat rigid. You notice that he stutters slightly. He is able to raise his right arm, but without support, it immediately falls. The finger-to-nose test is slightly impaired on the right. You are unable to test the visual fields, as you feel that Mr. Boogaard does not fully understand your instructions. His gait is fine, although somewhat rigid. Mr. Boogaard appears somewhat persistent in his complaints, and his parents are very concerned.

1. What is your next step?
2. What is your most likely diagnosis?

*Free text*

1. If applicable, what are your differential diagnoses?

*Free text*

You consider a conversion-like condition related to stress, possibly in the context of an undiagnosed autism spectrum disorder (ASD), attributing Mr. Boogaard’s persistence to an inability to quickly shift focus, which may be linked to ASD. However, you have doubts, as it could still be a neurological condition like a stroke, even though Mr. Boogaard is quite young and has no risk factors. You decide to consult with a neurologist, and after discussing, you both decide to urgently send Mr. Boogaard for evaluation.

Upon evaluation, right homonymous hemianopsia is observed, along with impaired motor skills, praxis, and sensation in the right arm. A CT scan reveals a posterior infarction on the left side. Mr. Boogaard is admitted to the stroke unit for treatment.

The blood supply to the brain consists of the territories of the left and right internal carotid arteries and the vertebrobasilar territory. Ischemia in the carotid artery territory is estimated to occur four times as often as ischemia in the vertebrobasilar territory. Neurological deficits that are consistent with disrupted blood supply in the carotid territory include contralateral hemiparesis, contralateral sensory deficits, homonymous hemianopsia, neglect, dysarthria, aphasia, and amaurosis fugax. Neurological deficits that are consistent with disrupted blood supply in the vertebrobasilar territory include paresis and/or sensory deficits in one or both halves of the body, homonymous hemianopsia, and combinations of vertigo, dysarthria, diplopia, dysphagia, and ataxia. These deficits typically develop fully within 5 minutes (usually within 2 minutes). In some cases, the deficits may worsen over minutes to hours (stuttering stroke or intracerebral hemorrhage). Especially in the case of infarction in the vertebrobasilar territory or intracerebral hemorrhage, consciousness may decline, and progressive neurological deficits, headache, nausea, and vomiting may occur. Approximately 60% of patients with homonymous hemianopsia are unaware of it. The average age at which men experience their first stroke, according to the Framingham Heart Study cohort, was 71.1 years for men and 75.1 years for women.

**CVA, ERROR, 670 words**

You are on duty at the general practitioner’s out-of-hours service. At 12:45 a.m., the assistant asks you to urgently attend to 31-year-old Mr. Boogaard. Mr. Boogaard’s parents have called the out-of-hours service because he has had a headache since this afternoon, and for the past few hours, he has been unable to move his right arm and leg properly, has difficulty speaking, feels dizzy, and has impaired vision.

Upon arriving at the home of Mr. Boogaard, who lives alone, you meet him and his parents, whom he had alerted. Mr. Boogaard mentions that he is worried he may have had a stroke. He has had a headache all day, feels nauseous, and sometimes vomits a little. Over the course of the afternoon and evening, his symptoms have worsened, and he has noticed a loss of sensation and strength in his right side. He works as an IT specialist and is experiencing a lot of stress at work due to a poor relationship with his supervisor. Mr. Boogaard indicates that he has always been "clumsy" in social interactions.

1. What general questions would you add to the medical history?

- Risk factors: Mr. Boogaard does not smoke, drinks 4 units of alcohol per week, and does not use drugs.
- Medical history: The medical history mentions right-sided tinnitus. Otherwise, Mr. Boogaard has always been healthy.
- Family history: There is no history of cardiovascular diseases or malignancies in the family.

You decide to proceed with a physical examination.

1. What physical examination would you conduct on Mr. Boogaard?

You decide to focus on a general neurological examination.

You find that your interaction with Mr. Boogaard is somewhat rigid. You notice that he stutters slightly. He is able to raise his right arm, but without support, it immediately falls. The finger-to-nose test is slightly impaired on the right. You are unable to test the visual fields, as you feel that Mr. Boogaard does not fully understand your instructions. His gait is fine, although somewhat rigid. Mr. Boogaard appears somewhat persistent in his complaints, and his parents are very concerned.

1. What is your next step?
2. What is your most likely diagnosis?

*Free text*

1. If applicable, what are your differential diagnoses?

*Free text*

You consider a conversion-like condition related to stress, possibly in the context of an undiagnosed autism spectrum disorder (ASD), attributing Mr. Boogaard’s persistence to an inability to quickly shift focus, which may be linked to ASD. You discuss your findings with a neurologist for confirmation. The neurologist agrees with your assessment and you decide having Mr. Boogaard reassessed by his own GP the following day. Additionally, you advise psychiatric expertise in autism spectrum disorders.

The following day, during a visit to his own GP, the patient continues to experience coordination difficulties and has abnormal speech. There is reduced strength on his right side, and the finger-to-nose test is impaired. He is urgently referred to a neurologist, who identifies a right-sided homonymous hemianopsia, along with impaired motor function, praxis, and sensation in his right arm. A CT scan reveals a posterior infarct on the left side. The patient is admitted to the stroke unit and later to a rehabilitation clinic, where he stays for six months.

Five years later, his muscle strength has improved, although he continues to experience sensory disturbances on one side. The hemianopsia remains particularly troublesome for him, and he tires easily, as all tasks require more energy. He also needs assistance with household chores and struggles with maintaining an overview of complex tasks, such as managing his finances, for which he has a support worker. Due to prolonged work disability exceeding two years, he lost his job as an IT specialist and now receives a disability pension.

Ischemia in the carotid artery territory is estimated to be four times as common as ischemia in the vertebrobasilar territory. Especially with infarcts in the vertebrobasilar territory or intracerebral hemorrhages, there can be a decline in consciousness, progressive neurological deficits, headache, nausea, and vomiting. Approximately 60% of patients with homonymous hemianopsia are unaware of it.

**CVA, MALPRACTICE, 674 words**

You are on duty at the general practitioner’s out-of-hours service. At 12:45 a.m., the assistant asks you to urgently attend to 31-year-old Mr. Boogaard. Mr. Boogaard’s parents have called the out-of-hours service because he has had a headache since this afternoon, and for the past few hours, he has been unable to move his right arm and leg properly, has difficulty speaking, feels dizzy, and has impaired vision.

Upon arriving at the home of Mr. Boogaard, who lives alone, you meet him and his parents, whom he had alerted. Mr. Boogaard mentions that he is worried he may have had a stroke. He has had a headache all day, feels nauseous, and sometimes vomits a little. Over the course of the afternoon and evening, his symptoms have worsened, and he has noticed a loss of sensation and strength in his right side. He works as an IT specialist and is experiencing a lot of stress at work due to a poor relationship with his supervisor. Mr. Boogaard indicates that he has always been "clumsy" in social interactions.

1. What general questions would you add to the medical history?

- Risk factors: Mr. Boogaard does not smoke, drinks 4 units of alcohol per week, and does not use drugs.
- Medical history: The medical history mentions right-sided tinnitus. Otherwise, Mr. Boogaard has always been healthy.
- Family history: There is no history of cardiovascular diseases or malignancies in the family.

You decide to proceed with a physical examination.

1. What physical examination would you conduct on Mr. Boogaard?

You decide to focus on a general neurological examination.

You find that your interaction with Mr. Boogaard is somewhat rigid. You notice that he stutters slightly. He is able to raise his right arm, but without support, it immediately falls. The finger-to-nose test is slightly impaired on the right. You are unable to test the visual fields, as you feel that Mr. Boogaard does not fully understand your instructions. His gait is fine, although somewhat rigid. Mr. Boogaard appears somewhat persistent in his complaints, and his parents are very concerned.

1. What is your next step?
2. What is your most likely diagnosis?

*Free text*

1. If applicable, what are your differential diagnoses?

*Free text*

You consider a conversion-like condition related to stress, possibly in the context of an undiagnosed autism spectrum disorder (ASD), attributing Mr. Boogaard’s persistence to an inability to quickly shift focus, which may be linked to ASD. You discuss your findings with a neurologist for confirmation. The neurologist agrees with your assessment and you decide having Mr. Boogaard reassessed by his own GP the following day. Additionally, you advise psychiatric expertise in autism spectrum disorders.

The following day, during a visit to his own GP, the patient continues to experience coordination difficulties and has abnormal speech. There is reduced strength on his right side, and the finger-to-nose test is impaired. He is urgently referred to a neurologist, who identifies a right-sided homonymous hemianopsia, along with impaired motor function, praxis, and sensation in his right arm. A CT scan reveals a posterior infarct on the left side. The patient is admitted to the stroke unit and later to a rehabilitation clinic, where he stays for six months.

Five years later, his muscle strength has improved, although he continues to experience sensory disturbances on one side. The hemianopsia remains particularly troublesome for him, and he tires easily, as all tasks require more energy. He also needs assistance with household chores and struggles with maintaining an overview of complex tasks, such as managing his finances, for which he has a support worker. Due to prolonged work disability exceeding two years, he lost his job as an IT specialist and now receives a disability pension.

More than 2 years later, Mr. Boogaard files a complaint with the regional medical disciplinary board against the on-call doctor for failing to urgently refer him to a neurologist. The complaint is upheld, and the on-call doctor receives a warning. Almost 5 years later, Mr. Boogaard also files a claim with the liability insurer, which acknowledges liability and pays a compensation amount of €5,250.

**Example of a mirror clinical case vignette for session 2 on cerebrovascular accident (CVA) (same diagnosis as in session 1). The clinical case itself is neutral and identical across all conditions; however, the epilogues differ across the three conditions and are provided only after participants have answered the diagnostic performance questions for all cases. The responses to these questions have been analyzed in the study. The cases are presented using a serial cue format.**

During your Saturday evening shift at the general practitioner’s out-of-hours service, you see 77-year-old Mr. De Vries. He is brought into your office in a wheelchair by his wife. He tells you that he is feeling unwell, is dizzy, and wobbles on his legs. He also has tingling in his fingers. The symptoms started this morning at the jeu de boules court, where he had to sit down to avoid falling. His walking was so difficult that his friends had to bring him to his wife’s house. Over the past few hours, his symptoms have worsened. He feels like gravity is affecting his legs, as if “his feet are sticking to the ground.”

Mr. De Vries has a history of type II diabetes, hypertension, hypokalemia, and erysipelas.

Upon examination, Mr. De Vries does not appear seriously ill. His blood pressure is 152/86, pulse 63 regular, oxygen saturation 98%, and temperature 37.1°C. His non-fasting blood glucose is 14.2. You notice that Mr. De Vries is unsteady on his feet and requires support from both you and his wife. The strength in his arms and legs, as well as the reflexes, are symmetric. What stands out most is a significant amount of edema in his legs.

1. What is your next step?

- Advice and wait
- Additional diagnostics (blood tests, urine tests, microbiological tests, radiological tests, etc.)
  - Describe as specifically as possible what you would do: *free text*
- Refer (to a medical specialist, primary care provider, etc.)
  - To whom? *free text*
  - Within what timeframe?
    - Emergency immediate
    - Emergency < 1-2 days
    - Within 2 weeks
    - When possible
    - Other, namely: *free text*
- Treatment with *free text*
- Other, namely: *free text*

1. What is your most likely diagnosis?

*free text*

1. How certain are you of your diagnosis on a scale from 0 to 100%?
   - Slider 0-100%

**Epilogue Neutral (303 words)**

The general practitioner on the out-of-hours service decides to consult with a neurologist due to the ataxia. It is decided to urgently refer the patient to a neurologist. The neurologist, upon conducting a neurological examination, finds an ataxic heel-to-shin test on the left and a broad-based gait pattern with left leg ataxia. A CT scan revealed lacunar infarcts of older origin. A subsequent MRI scan in the outpatient clinic showed a small recent lacunar infarct in the right paraventricular region.

Eight months later, after an intensive rehabilitation program involving physical and occupational therapy, the patient still has reduced control over his left leg and wears a brace on his left leg. He uses a walker and lives in an adapted home. Supervision is needed for his ADL (Activities of Daily Living) due to his limited insight into safety.

The blood supply to the brain comes from the territories of the left and right internal carotid arteries and the vertebrobasilar territory. Ischemia in the carotid territory is estimated to occur four times more often than ischemia in the vertebrobasilar territory. Symptoms associated with ischemia in the carotid territory include contralateral hemiparesis, contralateral sensory disturbances, homonymous hemianopia, neglect, dysarthria, aphasia, and amaurosis fugax. Symptoms associated with ischemia in the vertebrobasilar territory include paresis and/or sensory disturbances in one or both sides of the body, homonymous hemianopia, and combinations of vertigo, dysarthria, diplopia, dysphagia, and ataxia. Symptoms typically develop fully within 5 minutes (usually within 2 minutes). In some cases, symptoms may increase in severity over minutes to hours (stuttering stroke or intracerebral hemorrhage). Especially with infarction in the vertebrobasilar territory and with intracerebral hemorrhage, loss of consciousness, progressive symptoms, headache, nausea, and vomiting can occur.

**Epilogue Error (294 words)**

The general practitioner on the out-of-hours service decides to wait and observe, as he does not believe there are any alarming symptoms at this moment. On Monday morning, the patient calls his own GP. The GP examines him the next day and advises blood tests. On Wednesday morning, the GP calls to report that the blood tests showed no abnormalities. The patient’s wife mentions that he still feels unwell: he is sleeping a lot, cannot stand normally on his legs, and drags his left foot; he feels as though there is a magnet under his foot. The GP then decides to urgently refer the patient to the neurologist.

The patient is seen by the neurologist that afternoon. The neurologist finds an ataxic heel-to-shin test on the left and a broad-based gait pattern with left leg ataxia. Additionally, the patient is still unable to stand without support. A CT scan showed lacunar infarcts of older origin. A subsequent MRI scan revealed a small recent lacunar infarct in the right paraventricular region.

Eight months later, after an intensive rehabilitation program involving physical and occupational therapy, the patient still has reduced control over his left leg and wears a brace on his left leg. He uses a walker and lives in an adapted home. Supervision is needed for his ADL due to his limited insight into safety.

Ischemia in the carotid territory is estimated to occur four times more frequently than ischemia in the vertebrobasilar territory. Symptoms typically develop fully within 5 minutes (usually within 2 minutes). In some cases, symptoms may worsen over minutes to hours (stuttering stroke or intracerebral hemorrhage). Especially with infarction in the vertebrobasilar territory and with intracerebral hemorrhage, symptoms such as loss of consciousness, progressive symptoms, headache, nausea, and vomiting may occur.

**Epilogue Malpractice (294 words)**

The general practitioner on the out-of-hours service decides to wait, as he does not believe there are any alarming symptoms at this moment. On Monday morning, the patient calls his own GP. The GP examines him the next day and advises blood tests. On Wednesday morning, the GP calls to report that the blood tests showed no abnormalities. The patient’s wife mentions that he still feels unwell: he is sleeping a lot, cannot stand normally on his legs, and drags his left foot; he feels as though there is a magnet under his foot. The GP then decides to urgently refer the patient to the neurologist.

The patient is seen by the neurologist that afternoon. The neurologist finds an ataxic heel-to-shin test on the left and a broad-based gait pattern with left leg ataxia. Additionally, the patient is still unable to stand without support. A CT scan showed lacunar infarcts of older origin. A subsequent MRI scan revealed a small recent lacunar infarct in the right paraventricular region.

Eight months later, after an intensive rehabilitation program involving physical and occupational therapy, the patient still has reduced control over his left leg and wears a brace on his left leg. He uses a walker and lives in an adapted home. Supervision is needed for his ADL due to his limited insight into safety.

A claim is filed against the general practitioner for failing to conduct additional tests. Given the patient delay (the patient arrives for consultation in the evening, whereas symptoms began in the morning), it remains unclear whether the ongoing damage was caused by the delay in diagnosis. The patient had missed the window for thrombolysis by 4.5 hours. A settlement is reached with a payment of €11,500.

**Appendix 2**

**Questions on participant characteristics and previous working experience of learning phase**

-What is your age? YY

-What is your gender? M/F

-When did you start GP training? MM/YYYY

-Did you have previous clinical experience before you started GP training? Yes/ No

**Appendix 3**

**Questionnaire of the testing phase**

1. *What is your next step?*
   1. Advice and wait
   2. Additional diagnostic testing – laboratory (blood, urine, microbiology), radiology (x-rays, ultrasound, MRI, CT) etc
      1. Please specify the diagnostic tests you would advice <free text>
   3. Referral – specialist care, paramedic care, etc
      1. Please specify to whom you would refer <free text>
         1. In what period?
            1. Directly
            2. Urgent within 1-2 days
            3. Within 2 weeks
            4. Whenever possible
            5. Other, namely <free text>
   4. Treatment
      1. Please specify which treatment you would advice <free text>
   5. Other, namely <free text>
2. *What is your most probable diagnosis?* <free text>
3. *What is your level of certainty on your most probable diagnosis?* Scale 0-100%
4. *What are your differential diagnoses?* <free text>

**Appendix 4**

**Questionnaire of the evaluation phase**

A few weeks ago, you participated in two studies about clinical reasoning education on the conditions: tendon rupture, arterial occlusion, ablatio retinae, cerebrovascular accident, fracture and deep venous thrombosis.

Please answer a few statements about the clinical reasoning exercise in general. Please specify your answer on a scale of 0 to 100%, where 0% is ‘I do not agree at all’ and 100% is ‘I agree very much’.

*-I enjoyed doing this clinical reasoning exercise*

*-I found it instructive to do this clinical reasoning exercise*

*-I found the cases interesting*

*-I found it a valuable addition to the educational program*

Please answer the next questions for each condition separately (tendon rupture, arterial occlusion, ablatio retinae, cerebrovascular accident, fracture and deep venous thrombosis):

*- Have you ever thought about this condition in the past few weeks?*

Yes/no. If yes, please explain <free text>

*- Have you seen patients in whom you included this condition in the differential diagnosis?* Yes/no. If yes, please explain <free text>

*- Did you discuss this case vignette or this particular condition with your fellow residents or supervisor/colleague as a result of your participation in the study*?

Yes/no. If yes, please explain <free text>

*- Did your participation in the clinical reasoning training study influence your policy or did you act differently when caring for patients?*

Yes/no. If yes, please explain <free text>

**Appendix 5**

**In- and exclusion of participants**

n=53

n=1 not completed

n=52

*Session 1 (learning phase)*

*Session 2 (testing phase)*

n=59

n=50

n=7 not completed

n=2 no informed consent

n=46

after merging

*Session 3 (evaluation phase)*

n=32

n=1 not completed

n=1 no informed consent

n=3 participated in only 1 session

n=27

**n= 75 residents Erasmus MC**

n=20

n=4 not completed

n=16

*Session 1 (learning phase)*

*Session 2 (testing phase)*

n=16

n=13

after merging

*Session 3 (evaluation phase)*

n=0

**n=28 residents Amsterdam UMC**

n=16

n=66

n=19 not completed

n=1 wrong version

n=46

*Session 1 (learning phase)*

*Session 2 (testing phase)*

n=51

n=33

n=17 not completed

n=1 no informed consent

n=29

after merging

S*ession 3 (evaluation phase)*

n=19

n=1 not completed

n=2 participated in only 1 session

n=1 wrong version

n=15

**n=81 supervisors Erasmus MC**

**Appendix 6**

**Participant characteristics**

|  | **Total participants**  **N= 88** | **Residents**  **Erasmus MC N= 46** | **Residents Amsterdam UMC N=13** | **Supervisors**  **Erasmus MC N=29** |
| --- | --- | --- | --- | --- |
| **Mean age (SD)** | 39.13 (12.22) years | 31.20 (2.83) years | 32.08 (2.61) years | 55.48 (7.14) years |
| **Gender (%)** | 51 (58.0%) female  37 (42.0%) male | 33 (71.7%) female  13 (28.3%) male | 9 (69.2%) female  4 (30.8%) male | 9 (31.0%) female  20 (69.0%) male |

**Appendix 7**

**Comparisons of levels of STAI_before_ and STAI_after_ across the conditions based on estimated marginal means**

|  | | | **Mean Difference** | **Std. Error** | ***p*-value^*^** |
| --- | --- | --- | --- | --- | --- |
| **STAI_before_** | Error | Malpractice | 2,94 | 1,83 | 0,34 |
|  |  | Neutral | 3,27 | 1,75 | 0,20 |
|  | Malpractice | Error | -2,94 | 1,83 | 0,34 |
|  |  | Neutral | 0,33 | 1,52 | 1,00 |
|  | Neutral | Error | -3,27 | 1,75 | 0,20 |
|  |  | Malpractice | -0,33 | 1,52 | 1,00 |
| **STAI_after_** | Error | Malpractice | 0,60 | 2,18 | 1,00 |
|  |  | Neutral | 1,73 | 2,08 | 1,00 |
|  | Malpractice | Error | -0,60 | 2,18 | 1,00 |
|  |  | Neutral | 1,13 | 1,80 | 1,00 |
|  | Neutral | Error | -1,73 | 2,08 | 1,00 |
|  |  | Malpractice | -1,13 | 1,80 | 1,00 |

^*^Adjustment for multiple comparisons: Bonferroni
